# The Polish Version of the Resilience Scale 25: Adaptation and Preliminary Psychometric Evaluation

**DOI:** 10.3389/fpsyg.2021.668800

**Published:** 2021-05-14

**Authors:** Karol Konaszewski, Sebastian Skalski, Janusz Surzykiewicz

**Affiliations:** ^1^Faculty of Education, University of Bialystok, Bialystok, Poland; ^2^Institute of Psychology, Polish Academy of Sciences, Warsaw, Poland; ^3^Faculty of Philosophy and Education, Katholische Universität Eichstätt-Ingolstadt, Eichstätt, Germany; ^4^Faculty of Education, Cardinal Stefan Wyszynski University in Warsaw, Warsaw, Poland

**Keywords:** resilience, health, coping, resilience scale, validation

## Abstract

**Background:** The aim of the presented series of studies was to test the factor structure and assess the psychometric properties of the Resilience Scale 25 in the Polish population. It was developed and tested during the course of four independent studies analysing various aspects of the validation of the RS 25 questionnaire’s Polish version.

**Method:** Study 1 concerned the procedure for developing the Polish language version. Study 2 (*N* = 2716) consisted of reliability tests and a confirmatory factor analysis. In Studies 3 (*N* = 733) and 4 (*N* = 431), the validity was assessed by examining the relationship between resilience and the assessment of ego-resiliency, the risk of depression, styles of coping with stress, perceived stress, and satisfaction with life.

**Results:** The presented research results obtained using the measure indicate that it can be considered to be a reliable and valid research tool. A five-factor solution showed a good fit to the data: χ^2^/*df* = 12.85; RMSEA = 0.066 (low = 0.064; high = 0.068; 90% CI); GFI = 0.90; AGFI = 0.90. An assessment of the internal consistency was carried out on the basis of Cronbach’s alpha. The values achieved were satisfactory and indicate acceptable internal reliability of the questionnaire (0.89) and of the five dimensions: (1) purpose (0.65); (2) equanimity (0.65); (3) self-reliance (0.75); (4) perseverance (0.72); and (5) existential aloneness (0.66). In accordance with the predictions and earlier studies, resilience was correlated positively with ego-resiliency, a task-oriented style of coping with stress, and life satisfaction and negatively with perceived stress, the risk of depression, and an emotion-oriented coping style.

**Conclusion:** The Polish version of the RS 25 allows the assessment of the intensity of resilience as a general indicator and its five constituting dimensions. Such a measurement seems to be important from the perspective of assessing the role of an individual’s resources in clinical psychology, health psychology, and psychotherapy.

## Introduction

Research concerning resilience has increased significantly over the last few decades. More and more popular within educational practice are investigations regarding the potential impact of resilience on health, well-being, and quality of life (Bonanno et al., 2012; Liu et al., 2013; Sanders et al., 2015; Tempski et al., 2015; Cosco et al., 2016; Chen et al., 2018; Badu et al., 2020; Di Monte et al., 2020; Ungar and Theron, 2020). This interest results from the transition away from ‘deficit’ models looking for conditions of disease and psychopathology, and toward understanding healthy development in spite of risk factors along with a focus on strengths as opposed to weaknesses. Due to the growing interest in the strengths and what is ‘going in the right direction’ in terms of an individual’s development, interest has been placed on the construct of resilience, effective coping, and adaptation in the face of severe stress in life (Wyman et al., 1999; Rutter, 2013; Wagnild, 2013; Masten, 2014; Luthar, 2015).

In this area of research scientists used different tools to measure the level of resilience. Most had chosen to measure resilience with tools which focused on factors that can be defined as personality resources – for example: self-esteem, sense of coherence, self-efficacy, and hardiness. Others, in contrast, had used tools designed specifically for measuring the construct of resilience. Ahern et al. (2006) as well as Windle et al. (2011) reviewed the resilience measurement scales in all age groups and assessed their psychometric properties. They suggested that resilience, as a trait, was measured primarily with scales that obtained good psychometric properties: the CD-RISC Connor-Davidson Resilience Scale (Connor and Davidson, 2003), the DRS Dispositional Resilience Scale (Bartone, 2007), the ERS Ego-Resiliency Scale (Block and Kremen, 1996), and the Resilience Scale 25 (RS 25) (Wagnild and Young, 1993). In addition, researchers took advantage of resilience measures in different age groups in different contexts (Davydov et al., 2010; Zolkoski and Bullock, 2012).

Taking these aspects into consideration, it was decided to adapt the RS 25, which is a tool that possesses documented parametric properties and has been used by researchers in various cultural circles (Heilemann et al., 2003; Nygren et al., 2004; Nishi et al., 2010; Portzky et al., 2010; Abiola and Udofia, 2011). Compared with a study in which we confirmed the single-factor structure (shorter version) of the Resilience Scale 14 (RS 14) in three groups of youths and socially maladjusted youths (Surzykiewicz et al., 2019), in this study, we assessed the structure of the longer version of the RS 25 in the adult population. Additionally, we were able to indicate that the RS 25 allows us to assess the intensity of resilience, as a general indicator, and its constituting dimensions (e.g., perseverance, purpose, and equanimity). The RS 14 only allows the evaluation of the intensity of the overall resilience. The RS 25 also seems to be important from the perspective of assessing the role of an individual’s resources in clinical psychology, health psychology, and psychotherapy (Barzilay et al., 2020; Killgore et al., 2020; Walsh, 2020). Therefore, we undertook an assessment of the scale structure, indicating a solution that is appropriate for the group of adults. Such an extension allows for broader interpretations of the research results considering the detailed dimensions of resilience. To date, the RS 25 has not been validated in Polish conditions. However, due to the role of the scale (being reported in many studies and having confirmed psychometric properties), we decided to adapt it to Polish conditions.

### Resilience Scale 25 (RS 25)

The Resilience Scale 25 has gone through several stages of development over the years. The first scale studies were based on interviews conducted in 1987 with a selected group of 24 women who had adapted effectively despite serious life stressors. Research undertaken in the early nineties confirmed the reliability and validity of the RS 25. The research was conducted on a group consisting of students and graduates, youth, middle-aged and elderly people, caregivers of spouses with Alzheimer’s disease, mothers returning to work, and people living in municipal settlements (Wagnild and Young, 1993; Wagnild, 2009).

Initially, the RS consisted of 50 items. The scale was then reduced to 25 items reflecting five characteristic features of resilience. These are: *Purpose* (understanding that life is precious and meaningful, finding a direction in life); *Equanimity* (a balanced attitude towards one’s own life); *Self-Reliance* (belief in one’s own abilities and awareness of one’s limitations); *Perseverance* (ability to continue efforts, even after failing); and Independence and *Existential Aloneness* (consciously following one’s own path and accepting one’s own life). These five characteristics were identified by the authors as key elements of resilience (Wagnild and Young, 1993; Wagnild, 2009). In 1993, Wagnild and Young carried out a validation of the tool on a large group of people (*N* = 810). Using this group, the reliability and validity of the RS 25 were tested. An exploratory factor analysis allowed identification of the central foundations of the construct, presenting two interpretable factors of resilience: (1) *personal competences* that suggest self-reliance, independence, determination, ingenuity, and perseverance; and (2) *accepting self and life* in a manner that reflects adaptivity, flexibility and a balanced life perspective. This two-factor model explained 44.0% of the variance. Cronbach’s alpha coefficient for the entire scale was 0.91 (Wagnild and Young, 1993; Wagnild, 2003, 2009).

According to the conducted research, positive correlations have been noted between resilience measured with the RS 25 and self-efficacy, sense of coherence, satisfaction with life, healthy behaviours, mental well-being, ability to control stress, coping with stress focused on the task, and support of the environment, and negative strong correlations between resilience and depression, perceived stress, and anxiety (Beasley et al., 2003; Heilemann et al., 2003; Abolghasemi and Varaniyab, 2010; Davydov et al., 2010; Salazar-Pousada et al., 2010; Abiola and Udofia, 2011; Bonanno et al., 2012). The RS 25 has also been translated into many languages, the adaptations of which were used to demonstrate psychometric properties confirming the internal consistency, and reliability of the scale. Examples are: in Russian (Aroian et al., 1997); in Spanish (Heilemann et al., 2003; Ruiz-Párraga et al., 2012); in Swedish (Nygren et al., 2004); in Italian (Girtler et al., 2010); in Dutch (Portzky et al., 2010); in Japanese (Hasui et al., 2009; Nishi et al., 2010); in Nigerian (Abiola and Udofia, 2011; Oladipo and Idemudia, 2015); in Chinese (Lei et al., 2012); and in German (Schumacher et al., 2005).

The research conducted tested the one-, two-, and five-factor structures of the RS 25. In a Dutch study of 3265 individuals (Portzky et al., 2010), the two-factor structure was maintained. It was found to explain 29.7% of the total variance of resilience (17.34% for Factor 1; 12.32% for Factor 2). In a study of Japanese students (*N* = 504) (Hasui et al., 2009), the one-factor and two-factor solutions were tested using a confirmatory factor analysis. This model was found to fit the data acceptably. The fit indicators of the two models were virtually identical. In the Swedish adaptation (*N* = 142; ages 19 to 85) (Nygren et al., 2004), a two-factor solution was also confirmed, explaining 36.6% of the variance for the RS 25. In a subsequent Swedish adaptation (*N* = 1719; ages 19 to 103) (Lundman et al., 2007), an exploratory factor analysis identified five factors, including all 25 items of the scale. Using this five-factor scale, the variance was 52.5%. To confirm the structure of the factors and the psychometric properties in Spanish studies, Ruiz-Párraga et al. (2012) conducted an exploratory factor analysis of a group of people with chronic musculoskeletal pain. These authors derived a single-factor solution using a modified 18-item scale (RS-18), explaining 52.43% of the total variance. In a study of psychology and nursing students conducted in Japan, one-, two-, and five-factor solutions were not obvious (Nishi et al., 2010). Thus, there was no strong evidence for a clear structure of the RS 25, justifying the need for more validity and reliability evidence. In the current studies, the one-factor, two-factor, and five-factor solutions were tested.

### Aim of the Research

The goal of this series of studies was to test the factor structure and assess the psychometric properties of the RS 25 in the Polish adult population. It was developed and tested over the course of four independent studies (each study using different samples) analysing various aspects of validation of the Polish version of the RS 25. Study 1 concerned the procedure for developing the Polish language version. Study 2 (*N* = 2716) consisted of reliability tests and a confirmatory factor analysis (CFA). We assessed the one-factor (Ruiz-Párraga et al., 2012), two-factor (Wagnild and Young, 1993; Nygren et al., 2004; Portzky et al., 2010), and five-factor [Lundman et al., 2007; Wagnild and Young, 1991 (unpublished data)] solutions proposed by the researchers and authors of the scale. In Studies 3 (*N* = 733) and 4 (*N* = 431), the validity was assessed by examining the relationship between resilience and the assessment of ego-resiliency, the risk of depression, coping with stress, perceived stress, and satisfaction with life.

### Study 1. Developing the Polish Language Version of the Resilience Scale 25

The procedure of adapting the RS 25 proceeded as follows. After obtaining the author’s consent to prepare the Polish version of the RS 25 questionnaire, the tool was translated into Polish by two independent translators under the supervision of a person having knowledge of research methodology and promotion of psychology of health. Developing the Polish language version of the tool proceeded in several stages, during which we were in constant contact with the original author (G. Wagnild). The translation of the scale was done according to accepted principles developed for the purposes of intercultural research (WHO Translation Methodology) based on the original English version.

Efforts were also made to provide a good fit of the Polish language version to the age group of persons who represented the population for whom the tool was translated. For that reason, an assessment of preliminary understanding of the Polish version of the scale was carried out in a group of 12 people aged 18 to 60. The translated version of the tool was handed out during a group interview. The respondents’ task was to circle ‘yes’ if the question were completely understood and ‘no’ if something were not understandable or if the question caused any doubts. The outcome resulted in no problems nor doubts in understanding the statements. Nevertheless, the original version was further compared with the two re-translations and precautionary changes were introduced into the Polish version in order to reflect more pointedly the intentions of the authors and the basic contents of individual items. As adjustments were made, care was taken to maintain correct use of the Polish wording and syntax. Then, in order to acceptation and as well as possible translation discrepancies, a panel of experts was put together, composed of principal investigators, the original translator and two experts in health psychology. The final Polish version of the RS 25 was approved by a panel of experts.

### Study 2. Confirmatory Factor Analysis (CFA)

#### Materials and Methods

##### Participants and procedure

The psychometric properties of the Polish version of the scale and descriptive statistics were developed based on the results obtained by the (*N* = 2716) group of participants (women *N* = 2216 and men *N* = 498; aged from 19 to 63 years (age *M* = 24.40; *SD* = 5.44). We recruited the sample among full-time and part-time students and alumni of the University of Bialystok. Respondents received a research survey (paper format) directly from the researcher, who explained the purpose of the research, informed them about the voluntary character of participation, and stressed that each person could withdraw from participation in the research at any moment. In accordance with the Helsinki Declaration a written consent was received from all participants. The research project was approved by the Ethics Committee of the Faculty of Pedagogy and Psychology at the University of Bialystok.

##### Materials

*Resilience Scale 25*. Resilience is understood as a positive personality trait that facilitates personal adaptation (meaning coping with change or misfortune). All 25 items are assessed by the respondent on a seven-point scale from one (disagree) to seven (definitely agree), with possible total scores ranging from 25 to 175. Results higher than 145 indicate high resilience; results from 121 to 145 indicate moderate resilience; and results below 120 indicate low resilience (Wagnild and Young, 1993; Wagnild, 2009).

##### Data analysis

Subsequently, the confirmatory factor analysis (CFA) was applied to determine the factor structure. Analysis of structural equations was completed with the use of the AMOS program. Model parameters were estimated utilizing the maximum likelihood method. In order to assess the correctness of fitting the model to the data, the GFI (*goodness of fit index*), the AGFI (*(adjusted) goodness of fit*), RMSEA (*root-mean-square error of approximation*), and chi-square test (χ^2^/*df*) were employed. A AGFI and GFI ≥ 0.90 value indicates good and adequate adjustment of the model to the data (Hu and Bentler, 1999). Values χ^2^/*df* < 2 also suggest a good fit of the model to the data. A RMSEA < 0.08 value can also be interpreted as a good fit to the data (Kline, 2015; Byrne, 2016). Model refinement: often, a model’s fit indices may approach the abovementioned thresholds but not come close enough to be considered satisfactory. In such a case, minor adjustments to the relationships in the model can be made and the model can then be retested. The determination of which adjustments to make can be guided by the use of modification indices, which provide an estimate of the improvement in model fit that will occur by adding a given relationship, including direct paths and correlations (Schumacker and Lomax, 1996). The model will only be modified after an initial analysis if the modification meets the statistical criteria and fits the theoretical understanding of the RS 25. When modifications are added to a model, the model will be rerun and interpreted with the new fit indices.

The reliability of the RS 25 questionnaire was calculated using the Cronbach’s α coefficient and Interclass Correlation Coefficients (ICC). We used Student’s *t-*test to assess potential gender and age differences in resilience. Effect sizes were evaluated with Cohen’s *d*. Effects with *d* = 0.20 to 0.50 were interpreted as small, effects with *d = 0*.50 to 0.80 were considered medium and effects with *d* > 0.8 were considered large (J. Cohen, 1992). The Item Difficulty Index (IDI) was used to assess the ceiling and floor effects. Data granulation was concluded when over 50% of the respondents gave the same answer. A ceiling effect was observed when IDI > 0.8; the floor effect occurred when IDI < 0.2.

#### Results

##### Item analysis

Table 1 shows the basic descriptive statistics for the analysed items and the Item Difficulty Index (IDI) indicator. The distribution of analysed items was not observed to differ significantly from a normal distribution. In addition, the IDI indicator did not confirm the existence of floor or ceiling effects in the data (only in the case of rs3 rs5 rs18 a slight ceiling effect has been determined). A frequency analysis for individual test items showed no problems with data granulation. By reason of these results, all items were included in further analyses.

**TABLE 1:** Descriptive statistics and the IDI value for individual test items.

	M	SD	S	K	IDI
item1	5.36	1.22	−0.71	0.31	0.76
item 2	5.58	1.12	−0.82	0.99	0.78
item 3	5.81	1.26	−1.11	0.91	0.83
item 4	5.60	1.25	−0.75	0.23	0.80
item 5	5.94	1.20	−1.34	1.85	0.84
item 6	5.08	1.50	−0.64	−0.10	0.73
item 7	4.33	1.56	−0.26	−0.60	0.62
item 8	4.84	1.59	−0.57	−0.33	0.70
item 9	5.17	1.36	−0.75	0.31	0.73
item 10	5.10	1.37	−0.62	0.09	0.72
item 11	3.56	1.85	0.22	−1.03	0.52
item 12	4.89	1.33	−0.43	0.04	0.70
item 13	5.11	1.38	−0.53	−0.07	0.72
item 14	4.87	1.56	−0.51	−0.40	0.69
item 15	5.67	1.38	−1.06	0.75	0.81
item 16	5.53	1.36	−0.86	0.32	0.80
item 17	4.70	1.61	−0.42	−0.53	0.68
item 18	5.96	1.02	−1.16	1.96	0.84
item 19	5.41	1.23	−0.68	0.35	0.77
item 20	5.40	1.25	−0.85	0.88	0.76
item 21	5.58	1.56	−1.08	0.49	0.79
item 22	4.23	1.58	−0.07	−0.65	0.61
item 23	5.29	1.10	−0.51	0.40	0.75
item 24	5.03	1.40	−0.634	0.01	0.72
item 25	4.91	1.84	−0.556	−0.78	0.71

*M, average; SD, standard deviation; S, skewness; K, kurtosis; IDI, Item Difficulty Index/ceiling and floor effect.*

##### Assessment of the RS 25’s structure and reliability

We applied a confirmatory factor analysis in order to evaluate the validity of the RS 25 structure. We have assessed a one-factor, two-factor and five-factor solution. Table 2 presents the fit index for the assessed models.

**TABLE 2:** Fit index for assessed models.

	χ ^2^	χ ^2^/*df*	GFI	AGFI	RMSEA
1. Model one-factor	5901.83*	21.41	0.82	0.78	0.089 [low 0.087; high 0.091]
2. Model two-factors	5841.23*	21.31	0.84	0.82	0.081 [low 0.079; high 0.083]
3. Model five-factors	5044.10*	19.03	0.86	0.83	0.078 [low 0.076; high 0.080]
4. Model five-factors (modifications: 4 with 15 and 3 with 5)	3380.02**	12.85	0.90	0.90	0.066 [low 0.064; high 0.068]

*p < 0.001; **p < 0.05.

First, the one-factor model was tested. The second two-factor model was verified, in which two areas of resilience were defined without any modifications: (1) *personal competences* and (2) *self and life acceptance*. The third model, including no modifications, included a five-factor solution in which the five dimensions of resilience were considered: *(1) Purpose, (2) Equanimity, (3) Self-Reliance, (4) Perseverance*, and *(5) Existential Aloneness.* In the last, the five-factor solution was tested, taking into consideration the modification indicators.

**FIGURE 1:**
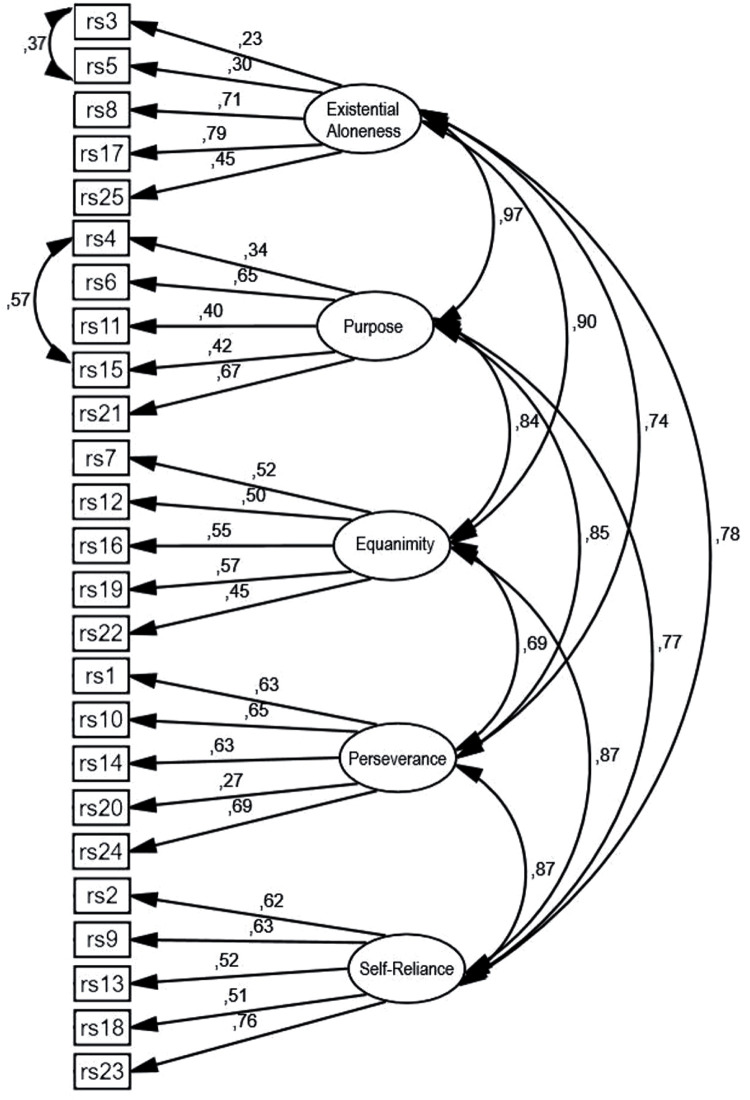
Five-factor resilience model with two modifications.

The first and second models (one-factor and two-factor solutions) turned out to be unacceptably fitted to the data; the analysed RMSEA, AGFI, and GFI fit values were unsatisfactory. The five-factor solution was also not well fitted to the data; the values of the GFI and AGFI indicators were unsatisfactory, but the RMSEA value was acceptable. Thus, in the fourth model, modification indices were used to detect the redundant items. We examined the modification indexes and identified two pairs of items (items: 4–15 and 3–5) that shared the remaining variance. We set these two correlated errors to be “free parameter estimates” and respecified the measurement model. The modified model proved to be well suited to the data, as indicated by the values of the RMSEA, AGFI, and GFI. In this manner, the five-factor structure was confirmed. Figure 1 shows a five-factor model with two modifications.

Cronbach’s alpha coefficient was used to examine the obtained RS 25 index. The reliability of the created factor was at 0.89 for the entire population and confirms its high consistency. Due to the fact that each of the subscales consists of only 5 statements for each factor, the reliability should be considered satisfactory: (1) Purpose 0.65, (2) Equanimity 0.65, (3) Self-Reliance 0.75, (4) Perseverance 0.72, and (5) Existential Aloneness 0.66. The generally accepted rule is that α of 0.6–0.7 indicates an acceptable level of reliability (Hulin et al., 2001; Taber, 2018). Intraclass Correlation Coefficients (ICC) estimates for 5 scales were moderate: (1) Purpose 0.19, (2) Equanimity 0.22, (3) Self-Reliance 0.32, (4) Perseverance 0.32, and (5) Existential Aloneness 0.27. The correlation of individual items with the scales was high (>0.30). Only for the perseverance item rs20 scale was it correlated at a low level (*r* = 0.16).

##### Descriptive statistics of RS 25 and relations based on sex and age

In the study group, the average result was *M* = 129.09; *SD* = 19.07 (min 43 – max 174). In addition, two distribution measures (skewness −0.46 and kurtosis 0.09) showed that the distribution of the formed indicator did not differ from the normal distribution. By means of the Student’s *t*-test analysis, it was examined whether there is a relation between the created RS 25 factor and sex. It was not confirmed that women (*M* = 129.09; *SD* = 18.80) differed from men (*M* = 129.06; *SD* = 20.26) in the level of the RS 25 index [*t*(2714) = −8.54, *p* > 0.05]. Correlation between resilience with age (*r* = 0.15; *p* < 0.001) was positive and weak.

### Studies 3 and 4. Convergent and Divergent Validity of the RS 25

#### Aim of the Studies

The aim of Studies 3 and 4 was to determine the validity of the RS 25 (the data were collected in the next studies, after confirming the scale structure and its internal consistency). To determine the convergent validity of the RS 25, we analysed the correlations among the RS 25 and ego-resiliency, life satisfaction, and coping styles (task and emotion). In line with the previous results, we expected positive relationships between the RS 25 and different resilience measures (Xiaonan and Jianxin, 2007), life satisfaction (Abolghasemi and Varaniyab, 2010; Tempski et al., 2015), and task-oriented coping (Beasley et al., 2003; Chen, 2016; Craparo et al., 2018). Furthermore, we expected a negative relationship between the RS 25 and perceived stress (Abolghasemi and Varaniyab, 2010; Beutel et al., 2017), risk of depression (Salazar-Pousada et al., 2010; Burns et al., 2011), and emotion–task coping (Gloria and Steinhardt, 2016). To determine the divergent validity of the RS 25, we analysed the correlations among the RS 25 and avoidance-oriented coping (Konaszewski et al., 2019).

#### Materials and Methods

##### Participants and procedure

Study 3 was conducted with 733 students of the Faculty of Education at the University of Bialystok, both full-time (*N* = 570) and part-time (*N* = 163), aged from 19 to 49 years (age *M* = 23.43, *SD* = 4.99), including 631 women and 102 men. Full-time and part-time students filled in the research survey during a lecture. Having received information about the research, data confidentiality, the voluntary nature of participation, and the possibility of withdrawing from the research at any moment, the respondents provided their informed consent. They completed the RS 25, the Coping Inventory for Stressful Situations (CISS), and the Ego-Resiliency Scale (ERS).

Study 4’s participants were 431 full-time (*N* = 350) and part-time (*N* = 81) students at the University of Bialystok, aged from 20 to 60 (age *M* = 25.32, *SD* = 7.40), consisting of 343 women and 88 men. The research was conducted among students of the Faculty of Education (78%) and the Faculty of Law (22%). The procedure was identical to that of Study 3. The participants completed the RS 25, the Kutcher Adolescent Depression Scale (KADS), the Perceived Stress Scale (PSS), and the Satisfaction with Life Scale (SWLS).

##### Measures

*Resilience.* The Polish version of RS 25 tested in Study 2 was used to assess resilience.

*Coping with stress*. To measure the styles of coping with stress, the Coping Inventory for Stressful Situations (CISS; Endler and Parker, 1994), in the Polish adaptation of Szczepanik, Wrześniewski, and Strelau, was employed. The scale includes 48 statements geared to determine which coping strategies a person chooses in a stressful situation. It entails the measuring of three basic styles of coping with stressful situations: (1) task-oriented style (TOS) implies purposeful and decisive focus on solving the problem and trying to make changes in the situation perceived as stressful; (2) emotional style (EOS) involves emotional responses – preoccupation with oneself, fantasizing, self-blaming; and (3) avoidance-oriented style (AOS) assumes the occurrence of an activity aimed at avoiding the problem, escaping from it without trying to solve it. The respondent determines the frequency with which the given action takes place in difficult and stressful situations on a 5-point scale (1 – never, 2 – very rarely, 3 – sometimes, 4 – often, 5 – very often). In research, the reliability of individual subscales in different age groups was, respectively: task style α = < 0.84; 0.90 >, emotional style α = < 0.86; 0.89 > and avoidance style α = < 0.78; 0.81 > (Strelau et al., 2005).

*Ego-Resiliency Scale* (ERS (Block and Kremen, 1996), in the Polish adaptation of Kaczmarek). In the validation studies of the original, it was determined that these items have a diagnostic value for ego-resiliency understood to be the ability to adjust the level of control over impulses to the requirements of the situation. The scale consists of 14 test items. The respondents answer on a 4-point scale, the extremes of which are 1 (does not apply at all) to 4 (refers very much). The result is a number between 13 and 52 with higher numbers representing greater intensity of the examined feature. The scale had a satisfactory internal consistency (α = 0.78) and high test-retest reliability (*r* = 0.89) (Kaczmarek, 2011).

*The Satisfaction with Life Scale* (SWLS) by Diener et al. (1985) assesses the sense of life satisfaction. This scale is intended for examining adults, both healthy and sick. This tool consists of five statements assessed on a 7-level point scale which constitutes the degree of satisfaction with one’s own life. The results range from 5 to 35 points. The higher the score, the greater the sense of life satisfaction. The author of the Polish adaptation is Juczyński. The SWLS reliability index (Cronbach’s alpha) was 0.81 (Juczyński, 2012).

*Kutcher Adolescent Depression Scale* [KADS (Brooks et al., 2003)]. The authors of the Polish scale are Mojs et al. (2015). The KADS is a widely used screening tool for assessing the risk of depression in youth and young adults. The respondents, using a 0– 3 scale, indicate whether they experience these emotions and thoughts rarely, sometimes, often, or always. A score equal to or greater than 6 indicates a risk of depression. The reliability of the scale calculated with the α-Cronbach coefficient was at *α* = 0.82 (Mojs et al., 2015).

*Perceived Stress Scale* (PSS; Cohen et al., 1983) in the Polish adaptation of Juczyński and Ogińska-Bulik (2012). The PSS consists of 10 items that assess perceived stress. The items are scored on a 4-point scale. The results range from 0 to 40 points. The PSS reliability index (Cronbach’s alpha) was 0.86. The scale had a high test-retest reliability (*r* = 0.90) (Juczyński and Ogińska-Bulik, 2012).

#### Results

RS 25 strongly and positively correlated with ego-resiliency (*r* = 0.60; *p* < 0.001), life satisfaction (*r* = 0.61; *p* < 0.001), and task-oriented style (*r* = 0.57; *p* < 0.001). Relation between resilience with perceived stress (*r* = −0.54; *p* < 0.001) and risk of depression (*r* = −0.51; *p* < 0.001). was strong and negative. Moreover, the relationship between resilience and emotion-oriented coping (*r* = −0.47; *p* < 0.001) was moderate and negative. The relation between resilience and the avoidance style (*r* = 0.03; *p* > 0.05) was irrelevant. The correlations between task-oriented style and existential aloneness (*r* = 0.44; *p* < 0.001), purpose (*r* = 0.43; *p* < 0.001), equanimity (*r* = 0.36; *p* < 0.001), perseverance (*r* = 0.53; *p* < 0.001) and the self-reliance (*r* = 0.51; *p* < 0.001) were significant (moderate or strong) and positive. The relations between emotion-oriented style and existential aloneness (*r* = −0.41; *p* < 0.001), purpose (*r* = −0.42; *p* < 0.001), equanimity (*r* = −0.35; *p* < 0.001), perseverance (*r* = −0.31; *p* < 0.001) and self-reliance (*r* = −0.37; *p* < 0.001) were significant (strong or moderate) and negative. The relations between avoidance-oriented style and existential aloneness (*r* = 0.02; *p* > 0.05), purpose (*r* = 0.02; *p* > 0.05), perseverance (*r* = −0.04; *p* > 0.05) and self-reliance (*r* = 0.01; *p* > 0.05) were insignificant. Moreover, the correlation analysis revealed a significant very weak positive relation between equanimity (*r* = 0.09; *p* < 0.05) and avoidance-oriented style. Ego-resiliency was significantly (strong or moderate) and positively connected with existential aloneness (*r* = 0.50; *p* < 0.001), purpose (*r* = 0.50; *p* < 0.001), equanimity (*r* = 0.48; *p* < 0.001), perseverance (*r* = 0.40; *p* < 0.001) and self-reliance (*r* = 0.53; *p* < 0.001). A correlation analysis showed that the risk of depression was negatively (strong or moderate) associated with existential aloneness (*r* = −0.45; *p* < 0.001), purpose (*r* = −0.48; *p* < 0.001), equanimity (*r* = −0.43; *p* < 0.001), perseverance (*r* = −0.36; *p* < 0.001), and self-reliance (*r* = −0.37; *p* < 0.001). Satisfaction with life was positively (strong or moderate) associated with existential aloneness (*r* = 0.46; *p* < 0.001), purpose (*r* = 0.62; *p* < 0.001), equanimity (*r* = 0.49; *p* < 0.001), perseverance (*r* = 0.48; *p* < 0.001) and self-reliance (*r* = 0.45; *p* < 0.001). There was also a negative moderate relation between the perceived stress and the existential aloneness (*r* = −0.48; *p* < 0.001), purpose (*r* = −0.46; *p* < 0.001), equanimity (*r* = −0.48; *p* < 0.001), perseverance (*r* = −0.40; *p* < 0.001), and self-reliance (*r* = −0.39; *p* < 0.001) (Table 3).

**TABLE 3:** The relation between the RS 25 index and selected psychological questionnaires – values of Pearson’s correlation coefficients.

	ER	TOS	EOS	AOS	SwL	RoD	PS
	Study 3 (*N* = 733)				Study 4 (*N* = 431)		
RS 25	0.60***	0.57***	−0.47***	0.03	0.61***	−0.51***	−0.54***
Existential aloneness	0.50***	0.44***	−0.41***	0.02	0.46***	−0.45***	−0.48***
Purpose	0.50***	0.43***	−0.42***	0.02	0.62***	−0.48***	−0.46***
Equanimity	0.48***	0.36***	−0.35***	0.09*	0.49***	−0.43***	−0.48***
Perseverance	0.40***	0.53***	−0.31***	−0.04	0.48***	−0.36***	−0.40***
Self-Reliance	0.53***	0.51***	−0.37***	0.01	0.45***	−0.38***	−0.39***

*RS 25* resilience; *ER* ego-resiliency; *TOS* task-oriented style; *EOS* emotional-oriented style; *AOS* avoidance-oriented style; *SwL* satisfaction with Life; *RoD* risk of depression; *PS*, perceived stress.*p < 0.05; ***p < 0.001 (significant level).

## Discussion

The research team confirmed the Polish version of the RS 25 as a research tool that measures resilience understood as a trait of a given individual, which works in favor of the process of adaptation in difficult situations. The research results indicate that it can be considered a reliable and accurate research tool. By means of the confirmatory factor analysis, it was determined that the items measure the five-factor construct. The obtained data indicate that the Polish version of the RS 25 presents satisfactory psychometric properties. The goodness of fit values are close to the results obtained by authors of the original scale version. RS 25 allows for assessing the intensity of resilience as a general indicator and its five constituting dimensions. Such measurement seems important from the perspective of assessing the role of an individual’s resources in clinical psychology, health psychology and psychotherapy.

Meanwhile, the structure of the scale was assessed based on CFA. As in the case of studies conducted by the authors of the scale and Swedish research (Lundman et al., 2007) a five-factor solution was confirmed. The analysed five-factor model possesses good fit measures and, as a consequence, the proposed construct of ‘resilience’ exists in Polish conditions. Therefore, it can be concluded that the RS 25 is characterized by a good factor structure. In conclusion, the study supported the five-factor solutions proposed by the authors of the scale [Wagnild and Young, 1993; Wagnild and Young, 1991 (unpublished data)]. Assessing the internal consistency was carried out on the basis of Cronbach’s alpha. The obtained values were acceptable and indicate an acceptable internal reliability of the questionnaire and five dimensions.

The validity of the scale has been studied by analysing relations of resilience with ego-resiliency, styles coping with stress, assessment of the risk of depression, perceived stress, and life satisfaction. Our research confirms previous findings in terms of which resilience is positively associated with the ego-resiliency (Xiaonan and Jianxin, 2007). According to predictions and earlier studies, resilience was positively correlated with the task-oriented style of coping with stress and life satisfaction, and negatively correlated with the assessment of the risk of depression and emotional style. The relation between an avoiding style and resilience was not determined.

Similar results were obtained in research by Stratta and co-workers (Stratta et al., 2013), which also confirmed a positive relation between resilience and a task-oriented style, as well as a negative relation with the emotional style. There was no proven relation between resilience and an avoiding style. In the studies conducted also, the relationship between resilience and avoidant coping style was irrelevant. Avoidant style can also be analysed in terms of its two forms (engaging in substitute activities and seeking social contact). Only then was the positive relationship between resilience and social contact seeking confirmed, while the relationship between engaging in substitute activities was insignificant (Konaszewski et al., 2019). In the course of other studies, the relation between resilience and task-oriented strategies of coping was also confirmed, and negative with emotion-oriented strategies (Beasley et al., 2003; Chen, 2016; Craparo et al., 2018; Konaszewski et al., 2019). Our research also confirms previous findings, in terms of which resilience is also negatively associated with the perceived of stress and risk of depression, while positively with satisfaction with life (Beasley et al., 2003; Ahern et al., 2006; Abolghasemi and Varaniyab, 2010; Salazar-Pousada et al., 2010; Tempski et al., 2015).

The average RS 25 score in the presented study was 129.09, which is comparable to previous studies (Lundman et al., 2007; Hasui et al., 2009; Wagnild, 2009; Nishi et al., 2010; Losoi et al., 2013), and indicates moderate resilience in the study group. As in previous studies (Lundman et al., 2007; Losoi et al., 2013), we did not find a relation between sex and resilience. It turned out that the resilience increases with age and confirms the view of resilience to be a dynamic process that can be modified (Lundman et al., 2007; Losoi et al., 2013). The positive relation between age and resilience intensity is a commonly reporter phenomenon (Sun and Stewart, 2007). It is believed that life capital grows with age, which is caused by a broader repertoire of available coping strategies, higher self-esteem, more strongly perceived social support and the emotional development of individuals. These variables favor the development of resilience (Belgrave et al., 2000). Our results confirm the findings of previous studies (Ong et al., 2006, 2009) in which attention was paid to the dependence of resilience on age. In those studies, the age of the participants was taken into consideration as a moderator. It was determined that levels of resilience are more probable depending on the age of an individual, including differences between young adults and adults (Abolghasemi and Varaniyab, 2010; Haddadi and Besharat, 2010; Cénat and Derivois, 2014).

The high percentage of young participants is limitation of the external validity of our study. The generalizability of the results therefore is limited to a student population and further validation in other adult samples is required. This is supported by the reported significant effect for age.

Resilience is a key personality trait, responsible for adaptation in the face of severe stress in life (Masten, 2014; Luthar, 2015). The application of resilience measures enables the anticipation of future coping of individuals with traumatic events and well-being (Rutter, 2013; Wagnild, 2013). RS25 serves as a support for previous tools determining the conditions of healthy growth. The scale we developed is one of the first in Poland which, apart from the general resilience indicator, allows for assessing the intensity of its individual elements (dimensions). This will allow for a better understanding of the role of specific components of the construct in the adaptation to various stress situations, as well as for developing suitable methods of intervention. The questionnaire is applicable in research, in the form of a psychological interview, as well as in therapy work, where quick reporting of changes linked to the conducted intervention is required. In future studies, it would seem reasonable to analyse the impact of resilience on stress, while at the same time controlling other accompanying variables (e.g., emotion-oriented style). Finally, it should be noted that we did not assess the absolute validity of the scale in the study. We believe that resilience intensity may change as a result of life experiences and undertaken interventions.

## Data Availability Statement

The raw data supporting the conclusions of this article will be made available by the authors, without undue reservation.

## Ethics Statement

The studies involving human participants were reviewed and approved by the Ethics Committee of the Faculty of Pedagogy and Psychology at the University of Bialystok. The patients/participants provided their written informed consent to participate in this study.

## Author Contributions

KK, SS, and JS conceived and designed the study, analysed the data, wrote the manuscript, and interpreted the results, drafted the manuscript and read and approved the final version. KK and JS performed the study. All authors contributed to the article and approved the submitted version.

## Conflict of Interest

The authors declare that the research was conducted in the absence of any commercial or financial relationships that could be construed as a potential conflict of interest.

## Abbreviations

AGFI: adjusted goodness of fit
AOS: avoidance-oriented style
CD-RISC: Connor-Davidson Resilience Scale
CISS: Coping Inventory for Stressful Situations
CFA: confirmatory factor analysis
DRS: Dispositional Resilience Scale
EOS: emotional-oriented style
ER: Ego-Resiliency
GFI: goodness of fit index
ICC: Interclass Correlation Coefficients
IDI: Item Difficulty Index
KADS: Kutcher Adolescent Depression Scale
PSS: Perceived Stress Scale
RMSEA: root-mean-square error of approximation
RS 25: Resilience Scale 25
SWLS: The Satisfaction with Life Scale
TOS: task-oriented style.
